# Anterior Lumbar Interbody Fusion Versus Total Disc Replacement with a Viscoelastic Prosthesis for Single-Level Lumbar Degenerative Disc Disease in Young Adults: A Comparative Retrospective Cohort Study

**DOI:** 10.3390/jcm15197409

**Published:** 2026-09-24

**Authors:** Clément Jacquemin, Matthieu Campana, Jean-Etienne Castelain, Houssam Bouloussa, Vincent Challier, Soufiane Ghailane

**Affiliations:** 1 Department of Spinal Surgery Unit, Hôpital Privé Francheville, 24000 Périgueux, France; clement.jacquemin.bdx@gmail.com (C.J.); drcampana@hpdf.fr (M.C.); drcastelain@hpdf.fr (J.-E.C.); challier.vincent@gmail.com (V.C.); 2 Department of Orthopaedic Surgery, University of Missouri-Kansas City, 2301 Holmes Street, Kansas City, MO 64108, USA; houssam.bouloussa@uhkc.org

**Keywords:** anterior lumbar interbody fusion, total disc replacement, spine surgery, lumbar degenerative disc disease, clinical outcomes, LP-ESP prosthesis, smoking

## Abstract

**Background/Objectives**: The optimal surgical approach for patients with lumbar degenerative disc disease (DDD) remains a subject of ongoing debate, particularly when comparing anterior lumbar interbody fusion (ALIF) and total disc replacement (TDR). The primary objective of this study was to compare short-term outcomes between these two surgical approaches in terms of clinical efficacy, patient satisfaction, and safety profile. The secondary objective was to evaluate patient-specific variables associated with postoperative improvement in order to identify potential factors that may guide a personalized surgical strategy. **Methods**: This retrospective, single-center observational study included 87 patients who underwent single-level lumbar surgery using either an ALIF (*n* = 38) or TDR (*n* = 49) approach between January 2021 and May 2025. Eligible patients were younger than 45 years and had complete preoperative, intraoperative, and 1-year follow-up data. Demographic, clinical, preoperative, and intraoperative variables, as well as patient satisfaction and quality-of-life improvement, were collected, analyzed, and compared between groups. The primary between-group analysis used analysis of covariance (ANCOVA) with adjustment for baseline outcome values and prespecified confounders (age, sex, body mass index, smoking status, ASA class). Longitudinal effects were assessed using a linear model with treatment × time interaction and cluster-robust standard errors. A formal smoking × surgical-strategy interaction was tested in a multivariable regression on 12-month Oswestry Disability Index (ODI) change. Reporting followed the STROBE guidelines for observational studies. **Results**: Both groups showed substantial within-group improvement at all follow-up time points (all *p* < 0.05 except ALIF leg pain at 6 months; paired Wilcoxon test). Baseline-adjusted between-group comparisons (ANCOVA) showed a numerically modest early difference favoring TDR for Visual Analogue Score (VAS) low-back pain at 3 and 6 months (adjusted Δ = −1.46, 95% CI −2.63 to −0.30, *p* = 0.016 at 3 months; −1.35, 95% CI −2.63 to −0.07, *p* = 0.043 at 6 months), below the minimum clinically important difference of 2 points though neither survived Holm–Bonferroni correction for multiple comparisons. The formal treatment × time interaction test was not significant for any outcome (ODI *p* = 0.39; VAS back *p* = 0.29; VAS leg *p* = 0.06. Overall, 92% of patients in the ALIF group and 86% of patients in the TDR group reported being satisfied or very satisfied, and 60–72% of patients returned to work. Radiographic analysis in the TDR group showed maintained segmental mobility, with median range of motion of 4.0° (IQR 2.0–8.0°) overall (median 6.0°, IQR 4.0–8.5° at L4–L5; 3.0°, IQR 2.0–6.0° at L5–S1). A multivariable regression model on 12-month ODI change, adjusted for age, sex, BMI, ASA class, and baseline ODI, showed a highly significant smoking-burden × surgical-strategy interaction (dose–response smoker code × TDR coefficient = −9.47, 95% CI −15.6 to −3.3, *p* = 0.004): higher preoperative smoking burden was associated with reduced ODI improvement specifically in the TDR group (Spearman r = −0.41, *p* = 0.004) but not the ALIF group (Spearman r = +0.17, *p* = 0.32). **Conclusions**: This cohort study suggests that both ALIF and TDR were associated with substantial clinical improvement and high patient satisfaction at 1 year postoperatively; because treatment selection was not randomized, these findings should not be interpreted as evidence of comparative effectiveness. A modest early difference favoring TDR was observed for VAS low-back pain (below the minimum clinically important difference); no statistically significant between-group differences were demonstrated for any outcome after correction for multiple comparisons. The observed associations between 1-year ODI improvement and patient-specific variables require confirmation in larger independent cohorts. The finding of a significant smoking-burden × surgical-strategy interaction is an exploratory and hypothesis-generating and, if confirmed in prospective cohorts, may inform preoperative optimization strategies for TDR candidates.

## 1. Introduction

Lumbar degenerative disc disease (DDD) represents a major global health burden, affecting an estimated 3.6% of the world’s population and contributing substantially to years lived with disability [1,2]. The associated disability burden increased by 54% between 1990 and 2015, with annual costs in the United States alone exceeding USD 100 billion, two-thirds of which are attributable to productivity losses [3,4]. However, the appropriate management of lumbar DDD remains controversial, with growing concerns regarding the overutilization of spine surgery. Recent studies have documented substantial geographic variation in fusion rates as well as evidence of unnecessary procedures in patients who have not exhausted conservative treatment options or who lack clear surgical indications [5,6,7]. When conservative treatments, including physical therapy, pharmacologic management, and interventional pain procedures, fail to provide adequate relief after at least six months of structured care, surgical intervention may be considered in carefully selected patients with confirmed pathology and functional impairment. Therefore, better characterization of patients who achieve favorable postoperative outcomes is needed to refine surgical indications and guide patient selection.

Anterior lumbar interbody fusion (ALIF) has traditionally been considered a standard surgical option for the management of symptomatic lumbar DDD. By achieving solid arthrodesis, ALIF can reliably alleviate pain and restore spinal stability [6,7]. However, fusion fundamentally alters spinal biomechanics by increasing mechanical stress on adjacent segments, which may contribute to adjacent segment disease (ASD), reported in up to 30% of patients within 10 years [8,9]. Pseudarthrosis and the need for revision surgery may further compromise long-term outcomes, with clinical success rates reported as low as 45% in some cohorts [10,11].

Total disc replacement (TDR) has emerged as a motion-preserving alternative designed to maintain physiological spinal kinematics while avoiding the biomechanical complications associated with fusion. Multiple meta-analyses have demonstrated favorable outcomes for TDR, including clinically comparable patient-reported outcomes and reduced adjacent segment degeneration compared with fusion [12,13]. However, this technology continues to evolve, with newer prosthetic designs aiming to more closely replicate native disc biomechanics.

The LP-ESP (Elastic Spine Pad) lumbar disc prosthesis represents a recent advancement in TDR technology. This viscoelastic implant combines a microcellular silicone gel core with a polycarbonate-urethane ring attached to titanium endplates and is designed to preserve spinal mobility while replicating the shock-absorbing properties of a healthy disc [14]. The LP-ESP represents a design concept distinct from conventional articulated total disc replacements: rather than employing two or three components with articulated bearing surfaces, the LP-ESP is a monobloc viscoelastic device providing six degrees of freedom with an intended shock-absorption profile mimicking that of the native disc. Early prospective studies with 2- and 5-year follow-up have demonstrated significant improvements in pain and disability scores, with maintained range of motion and physiological center of rotation in 73% of patients [14,15,16]. However, comparative evidence demonstrating clinical superiority of the LP-ESP over other disc prostheses remains limited, and previous studies have not directly compared LP-ESP with lumbar fusion. Despite these promising results, comparative data evaluating LP-ESP against established surgical techniques remain limited. In particular, key functional outcomes such as return to work rates and validated comparisons with fusion procedures have not been adequately investigated.

In the context of rising concerns regarding unnecessary spine surgery, it is essential to demonstrate that surgical interventions whether fusion or motion-preserving procedures provide meaningful clinical benefit when appropriately applied to well-selected patients. Previous studies have reported substantial variability in patient selection criteria and surgical outcomes [6,7], underscoring the need for rigorous comparative research in homogeneous populations with clearly defined surgical indications. This study focuses exclusively on young, active patients with confirmed single-level DDD refractory to conservative management, representing an appropriate cohort in which to evaluate the efficacy of surgical treatment while minimizing the risk of including patients who may have improved without intervention.

Smoking is a well-established, modifiable risk factor for poorer outcomes after lumbar fusion surgery, including higher rates of pseudarthrosis, delayed bone healing, and reduced patient-reported improvement; a recent large systematic review and meta-analysis confirmed this adverse association across the fusion literature [17]. Whether smoking similarly affects outcomes after TDR has been far less studied. A study of the ProDisc prosthesis [18] suggesting that smokers can nevertheless achieve improvements comparable to nonsmokers; because these devices differ mechanically from the monobloc viscoelastic LP-ESP construction, comparative data specific to LP-ESP, and to whether the surgical strategy itself modifies the impact of smoking on postoperative recovery, remain scarce.

The primary hypothesis of this study was that, in young and active patients with symptomatic single-level lumbar DDD refractory to conservative treatment, TDR with the LP-ESP viscoelastic prosthesis produces different short-term clinical outcomes compared with ALIF. The primary objective was to compare clinical outcomes at 12 months after surgery using patient-reported measures (Oswestry Disability Index and visual analog scales for low-back and leg pain). Secondary objectives included evaluating the longitudinal evolution of these outcomes, patient satisfaction, return-to-work status, and radiographic outcomes, and identifying patient-specific factors associated with postoperative improvement. To our knowledge, this is the first study directly comparing ALIF with the LP-ESP viscoelastic prosthesis in a homogeneous young, working-age population. Both procedures were performed through the same standardized anterior retroperitoneal approach, so that the compared variable was the reconstruction strategy (fusion versus motion) rather than the surgical corridor.

## 2. Materials and Methods

### 2.1. Study Design

This retrospective single-center observational cohort study was conducted at a single spine surgery center between January 2021 and May 2025. The study was conducted and reported in accordance with the STROBE (Strengthening the Reporting of Observational Studies in Epidemiology) guidelines for cohort studies; a completed STROBE checklist is provided as Supplementary Materials. The study protocol was approved by the SOFCOT Institutional Review Board, and all participants provided written informed consent in accordance with the Declaration of Helsinki.

### 2.2. Patient Selection

Eligible participants were adults aged 45 years or younger at the time of surgery, as the study was specifically designed to investigate outcomes in a young, active, working-age population. The 45-year threshold is consistent with previous lumbar total disc replacement literature (Guyer et al., J Neurosurg Spine 2008) [19]. Inclusion required a diagnosis of symptomatic single-level lumbar DDD (defined as disc herniation, Modic type 1 endplate changes, or both,) that was refractory to at least 6 months of structured conservative management including physical therapy, pharmacologic treatment, and where appropriate interventional pain procedures. Complete preoperative, intraoperative, and 12-month follow-up data were required. the age criterion was therefore a predefined study population parameter, not a post hoc exclusion. Inclusion was similarly restricted to the L4-L5 and L5-S1 segments, reflecting the natural distribution of symptomatic lumbar DDD in young adults, in whom degenerative changes predominate at these two lower lumbar levels (Sääksjärvi et al., Spine 2020; Ruangchainikom et al., Asian Spine J. 2021), consistent with the levels most commonly treated in the published lumbar TDR literature (Scott-Young et al., Int J Spine Surg. 2022) [20,21,22].

Exclusion criteria were prior lumbar spine surgery at any level, multi-level pathology, contraindications to either surgical strategy (e.g., severe facet arthropathy or spondylolisthesis precluding TDR), or incomplete baseline or 12-month follow-up data (the primary endpoint); missing intermediate 3- or 6-month data did not lead to exclusion and were handled via available-case analysis. During the study period, 162 patients were assessed for eligibility, of whom 75 were excluded. Detailed exclusion counts by category are provided in Figure 1.

### 2.3. Choice of Surgical Strategy

The choice between ALIF and TDR was based on well-defined clinical and imaging criteria applied consistently by the operating surgical team, and included: (i) presence or absence of segmental instability on preoperative dynamic radiographs (segmental instability favored ALIF); (ii) severity of facet arthropathy (severe facet degeneration favored ALIF, as facet arthropathy is a relative contraindication to TDR); (iii) integrity of the posterior spinal column and facet joints (preserved posterior elements permitting TDR); (iv) presence of spondylolisthesis or foraminal stenosis requiring direct decompression (favoring ALIF); (v) patient-specific anatomic considerations including endplate morphology and disc height; and (vi) shared decision-making with the patient after presentation of the theoretical advantages and limitations of each option. This selection process is illustrated in Supplementary Figure S1.

### 2.4. Surgical Techniques

All procedures were performed under general anesthesia by senior spine surgeons using standardized surgical techniques. The anterior retroperitoneal approach used for both procedures carries specific risks, including vascular injury, visceral injury, sympathetic chain dysfunction, and retrograde ejaculation in male patients; because both arms shared the same surgical corridor, these risks are not expected to differentially bias the between-group comparison.

### 2.5. ALIF Versus TDR

**Anterior Lumbar Interbody Fusion (ALIF):**

Patients underwent ALIF via a retroperitoneal approach, with vascular mobilization performed by a vascular surgeon. After exposure of the anterior disc space, a complete discectomy was performed, including removal of the cartilaginous endplates and preparation of bleeding bony endplate surfaces. The disc space was distracted using appropriate trials, and endplate dimensions were measured. Structural interbody cages (Spineart SA, Plan-les-Ouates, Switzerland; Clariance SAS, Paris, France; titanium alloy) were selected based on disc height and endplate geometry. Cages were packed with demineralized bone matrix (DBM) and autologous iliac-crest bone graft obtained during discectomy, then positioned under fluoroscopic guidance to achieve optimal lordosis and anterior column support without the use of posterior instrumentation.

**Total Disc Replacement (TDR):**

The LP-ESP prosthesis (Spineway, Lyon, France) was implanted through a similar anterior retroperitoneal approach. After vascular mobilization and exposure, a complete discectomy was performed while preserving the posterior longitudinal ligament. The disc space was carefully prepared and distracted to restore physiological disc height. Prosthesis sizing was determined using trials that matched the sagittal and coronal dimensions of the disc space. The prosthesis consists of a monobloc viscoelastic core combining microcellular silicone gel with a polycarbonate-urethane annulus, bonded to titanium endplates without adhesive. The prosthesis was positioned centrally under fluoroscopic guidance. Immediate stability was confirmed by direct intraoperative assessment.

### 2.6. Postoperative Management

All patients followed a standardized rehabilitation protocol. Mobilization began on postoperative day 1 with physical therapy assistance. Patients were discharged once they were ambulatory and had adequate pain control with oral medications, typically within 1–3 days. No postoperative bracing was required; however, all patients followed similar activity restrictions during the first 6 weeks. All patients subsequently received structured physical therapy emphasizing core strengthening and functional restoration. Follow-up visits were scheduled at 3, 6, and 12 months postoperatively. The 12-month visit was mandatory as part of the study protocol and was completed by all included patients. Attendance at intermediate 3- and 6-month visits was per routine clinical scheduling: some surgeon-scheduled 6-month visits were not performed when the 3-month assessment had already demonstrated satisfactory recovery trajectory (Complete attendance is summarized in Supplementary Table S8). Attendance did not differ significantly between groups at any time point Supplementary Table S8), indicating that the reduced 6-month attendance reflected the scheduling pattern described above rather than a differential loss to follow-up between treatment arms. Baseline characteristics were similar between patients with and without 6-month follow-up data for most variables when stratified by group (age, sex, smoking status, ASA class, baseline ODI, VAS low-back pain, and VAS leg pain), with one exception: body mass index was significantly lower among TDR patients missing 6-month data (23.19 ± 3.22 vs. 26.69 ± 3.95 kg/m^2^, *p* = 0.012), with a similar non-significant trend in the ALIF group (29.99 ± 5.35 vs. 26.59 ± 4.28 kg/m^2^, *p* = 0.069). The proportion of ALIF patients among those missing 6-month data was similar to the overall cohort proportion (*p* = 0.35), arguing against a treatment-specific pattern of missingness overall (Supplementary Table S9).

### 2.7. Outcome Measures

**Primary Outcomes:**

Pain was assessed using the Visual Analog Scale (VAS) for low back pain and leg pain on a 0–10 scale, with 0 indicating no pain and 10 indicating the worst imaginable pain. Scores were recorded preoperatively and at 3, 6, and 12 months postoperatively. Functional disability was assessed using the Oswestry Disability Index (ODI), reported as a percentage score from 0% to 100%, with 0% indicating no disability and 100% indicating complete disability, at the same time points. Patient satisfaction was assessed at 12 months using a validated 5-point Likert-scale questionnaire ranging from “very dissatisfied” to “very satisfied,” along with patient-reported percentage improvement in overall health.

**Secondary Outcomes:**

Return to work was recorded as a binary outcome at 12 months, with documentation of full-time versus part-time return (this binary measure supports comparison of proportions returning to work but does not support claims about the timing or speed of occupational recovery). Complications were classified temporally as early (≤30 days) or late (>30 days). Perioperative parameters included operative time, estimated blood loss, and length of hospital stay.

### 2.8. Data Collection

Standing lateral lumbar radiographs were obtained at 12 months postoperatively in all patients, and standardized upright lateral flexion and extension radiographs were obtained in the TDR group. Radiographs were acquired with the patient in maximal voluntary flexion and extension using standardized positioning per hospital protocol.

Fusion assessment in the ALIF group: fusion was assessed at 12 months and was defined by the presence of all three of the following criteria: (i) bridging trabecular bone across the disc space visible on standing lateral radiographs; (ii) absence of implant subsidence (defined as >2 mm loss of postoperative disc height) or migration; and (iii) absence of segmental motion (<2°) on flexion-extension radiographs. Computed tomography (CT) was obtained when clinically indicated to confirm fusion status in equivocal cases.

Segmental range of motion measurement in the TDR group: segmental angles at the operated level were measured on lateral flexion and extension radiographs using the Cobb technique, drawing tangent lines along the superior and inferior endplates of the operated segment. Specifically, the segmental angle at L4–L5 was measured as the angle between the superior endplate of L4 and the inferior endplate of L5, and the segmental angle at L5–S1 was measured as the angle between the superior endplate of L5 and the superior endplate of S1. Segmental ROM was calculated as the arithmetic difference between the extension and flexion segmental angles. Measurements were performed by two experienced observers independently, using Synapse PACS software Logiciel version 7.4 (FUJIFILM Healthcare, Tokyo, Japan), blinded to clinical outcomes; discrepancies greater than 2° were resolved by consensus review. Inter-observer reliability was excellent (intraclass correlation coefficient > 0.92). Our analysis characterizes the segmental motion maintained at 12 months postoperatively. Prosthesis-specific radiographic assessment: implant loosening was defined as a radiolucent line > 2 mm around the prosthesis; migration as >3 mm axial or coronal displacement from initial postoperative position; subsidence as >2 mm loss of postoperative disc height. Systematic assessment for these findings was performed on all 12-month radiographs.

### 2.9. Statistical Analysis

Continuous variables are reported as median (interquartile range, IQR) for non-normally distributed data and as mean ± standard deviation (SD) when comparison to previously published values in mean ± SD format is helpful; categorical variables are reported as frequency (percentage). Baseline covariate balance between groups was assessed using standardized mean differences (SMDs), with values < 0.10 indicating adequate balance, 0.10–0.25 modest imbalance, and >0.25 substantial imbalance (Supplementary Table S4). Additional between-group analyses were performed in a propensity-score-matched cohort (25 pairs, 50 patients) as an internal consistency check on the multivariable-adjusted full-cohort analysis (Supplementary Table S5). Baseline continuous variables were additionally compared using the Mann–Whitney U test, and categorical variables using Fisher’s exact test, for descriptive purposes. All statistical tests were two-tailed with a significance threshold of α = 0.05. Multiple comparisons across the three primary outcomes (ODI, VAS low-back pain, VAS leg pain) at three time points (3, 6, 12 months) were adjusted using the Holm–Bonferroni method to control the family-wise error rate; both raw and Holm-adjusted *p*-values are reported for the primary between-group comparisons.

Within-group changes from baseline to each follow-up time point were assessed using paired Wilcoxon signed-rank tests. The primary between-group analysis for each outcome (ODI, VAS low-back pain, VAS leg pain) at each postoperative time point was performed using analysis of covariance (ANCOVA; linear regression of the outcome at follow-up on treatment group with adjustment for the baseline value of the outcome and prespecified covariates including age, sex, body mass index, smoking status, and ASA class). This approach directly accounts for baseline imbalance, particularly the between-group difference in preoperative VAS low-back pain, and is the recommended analysis for cohort studies with baseline imbalance (Vickers & Altman, BMJ 2001) [23]. Adjusted between-group differences are reported with 95% confidence intervals. Longitudinal effects were formally assessed by fitting a linear model on postoperative time points with fixed effects for treatment, time, treatment × time interaction, baseline outcome value, and covariates. Standard errors were computed using a cluster-robust (“sandwich”) estimator (Liang & Zeger, 1986) [24] with patient as the cluster to account for within-subject correlation of repeated measures. The treatment × time interaction *p*-value indicates whether the between-group difference changes significantly over the postoperative follow-up period. A sensitivity analysis using multiple imputation (m = 10 imputations under the missing-at-random assumption, chained equations) was performed to assess robustness of the 3- and 6-month between-group comparisons to missing intermediate follow-up data (Supplementary Table S6).

Minimum clinically important difference (MCID) responder proportions were computed at each time point using thresholds of ≥10-point ODI reduction and ≥2-point VAS reduction, and compared between groups using Fisher’s exact test.

A formal smoking × surgical-strategy interaction was tested in a multivariable linear regression model of 12-month ODI change (baseline − 12-month; positive = improvement), including main effects for treatment and smoker code, the smoker × treatment interaction term, and covariates (age, sex, BMI, ASA class, baseline ODI). Two operationalizations of smoking were tested: binary (any smoker vs. non-smoker) and dose–response (ordinal smoker code 0–3 (Supplementary Table S1), corresponding to non-smoker, light, moderate, and heavy smoker categories).

## 3. Results

### 3.1. Patient Demographics and Baseline Characteristics

A total of 87 patients met the inclusion criteria and were included in the final analysis: 38 patients underwent ALIF and 49 underwent TDR with the LP-ESP prosthesis. Baseline characteristics of both groups are presented in Table 1 and Supplementary Table S4, with standardized mean differences (SMDs) reported for balance assessment. Age, smoking status, baseline VAS leg pain, and segment level were well balanced between groups (all SMDs < 0.20). Modest to substantial imbalance was observed for sex (SMD = 0.445; 68.4% female in ALIF vs. 46.9% in TDR), body mass index (SMD = 0.405) and ASA class distribution. The distribution of DDD diagnoses was similar between groups, with most patients presenting with Modic type 1 endplate changes (ALIF: 78.9% vs. TDR: 61.2%). Most patients had L5–S1 involvement (ALIF: 71.1% vs. TDR: 69.4%), with the remainder treated at L4–L5. Preoperative ODI (median 42.7, IQR 35.0–53.5 in ALIF vs. 42.0, IQR 30.0–48.0 in TDR; SMD 0.227) and VAS leg pain (median 6.0, IQR 5.0–8.0 vs. 7.0, IQR 5.0–7.0; SMD −0.020) were similar. Baseline VAS low-back pain differed between groups (median 8.0, IQR 7.0–8.0 in ALIF vs. 7.0, IQR 6.0–8.0 in TDR; SMD 0.441, *p* = 0.035); this imbalance was accounted for in all subsequent ANCOVA analyses of VAS low-back pain outcomes.

### 3.2. Perioperative Outcomes

Operative parameters were comparable between groups (Table 2). Mean operative time was 64.9 min in the ALIF group versus 62.1 min in the TDR group (*p* > 0.05). Intraoperative blood loss was minimal and similar between groups (63.9 mL for ALIF vs. 66.8 mL for TDR; *p* > 0.05). Length of hospital stay was brief in both cohorts, with a mean duration of 1.5 days for ALIF and 1.6 days for TDR (*p* > 0.05).

Intraoperative complications were rare, occurring in only one patient in the ALIF group (2.6%). Early postoperative complications (≤30 days) were infrequent, with one event in the ALIF group and three events in the TDR group (ALIF: 2.6% vs. TDR: 6.1%). Late complications (>30 days to 12 months) occurred in three patients in the TDR group, including two cases of implant loosening and one secondary decompression, and in one patient in the ALIF group, who developed adjacent segment disease. Full case-level detail for the two TDR implant-loosening cases (timing of diagnosis, presenting symptoms, radiographic findings, management, revision status, and 12-month outcome) is provided in Supplementary Table S3. Between-group comparisons of complication rates (Fisher’s exact test) are reported in Table 2; given the limited power of this cohort to detect differences in relatively infrequent events, these *p*-values should be interpreted with caution and are not intended as evidence of equivalence between groups.

### 3.3. Clinical Outcomes: Pain and Disability

Both surgical approaches resulted in significant improvements in pain and functional disability at all follow-up time points (Table 3, Figure 2; within-group paired Wilcoxon test, all *p* < 0.05 except ALIF leg pain at 6 months, *p* = 0.15). Baseline-adjusted between-group comparisons (ANCOVA, adjusted for baseline outcome value, age, sex, BMI, smoking status, and ASA class) showed no statistically significant difference in ODI at any time point (3 months: adjusted Δ = −5.08, *p* = 0.20, Holm-adjusted *p* = 0.80; 6 months: adjusted Δ = −6.34, *p* = 0.16, Holm-adjusted *p* = 0.80; 12 months: adjusted Δ = +0.52, *p* = 0.90, Holm-adjusted *p* = 1.0). For VAS low-back pain, TDR showed a modest advantage below the minimum clinically important difference of 2 points at 3 months (adjusted Δ = −1.46, 95% CI −2.63 to −0.30, *p* = 0.016, Holm-adjusted *p* = 0.144) and 6 months (adjusted Δ = −1.35, 95% CI −2.63 to −0.07, *p* = 0.043, Holm-adjusted *p* = 0.344), with no significant difference at 12 months. For VAS leg pain, a borderline difference was observed at 3 months only (adjusted Δ = −1.39, 95% CI −2.76 to −0.02, *p* = 0.050, Holm-adjusted *p* = 0.350), with no significant differences at 6 or 12 months. After Holm–Bonferroni correction for the nine primary between-group comparisons (three outcomes × three time points), no comparison remained statistically significant, including the two raw-significant VAS low-back pain comparisons above; this is consistent with the non-significant treatment × time interaction test reported below for all three outcomes. The formal treatment × time interaction test was not statistically significant for any outcome (ODI *p* = 0.39; VAS low-back pain *p* = 0.29; VAS leg pain *p* = 0.06), indicating that the between-group difference did not change significantly over the follow-up period. MCID responder proportions (≥10-point ODI reduction, ≥2-point VAS reduction) were numerically higher for TDR on VAS outcomes in the early postoperative period but did not reach statistical significance and converged by 12 months (Supplementary Table S7). Unadjusted between-group comparisons at each time point (Mann–Whitney U test on raw ODI and VAS values, without baseline adjustment) are reported alongside in Supplementary Table S2.

#### 3.3.1. VAS Low-Back Pain

In the ALIF group, mean VAS low-back pain decreased from 7.8 ± 1.3 at baseline to 5.5 ± 2.4 at 3 months, 5.4 ± 2.2 at 6 months, and 4.8 ± 2.9 at 12 months (*p* < 0.0001 vs. baseline). The TDR group showed greater early reductions, with scores decreasing from 7.1 ± 1.6 at baseline to 4.4 ± 2.4 at 3 months, 4.0 ± 2.3 at 6 months, and 4.2 ± 2.5 at 12 months (*p* < 0.0001 vs. baseline at all follow-up time points). Between-group comparisons mean scores decreased over time in both groups. Baseline-adjusted between-group differences (ANCOVA) are reported in Section 3.3 main paragraph; neither the 3-month nor the 6-month VAS low-back pain comparison survived Holm–Bonferroni correction for the family of nine primary comparisons.

#### 3.3.2. VAS Leg Pain

Similar patterns were observed for VAS leg pain. In the ALIF group, mean scores decreased from 6.0 ± 2.4 at baseline to 3.9 ± 3.1 at 3 months, 4.2 ± 3.0 at 6 months, and 3.4 ± 3.3 at 12 months. In the TDR group, mean scores decreased from 6.1 ± 2.5 at baseline to 3.1 ± 2.9 at 3 months, 3.3 ± 2.9 at 6 months, and 3.7 ± 3.1 at 12 months. Although early mean leg-pain scores were numerically lower in the TDR group, the 12-month scores were similar between groups.

#### 3.3.3. Oswestry Disability Index

Functional disability improved significantly in both groups. In the ALIF cohort, mean ODI decreased from 44.6 ± 13.3% at baseline to 33.3 ± 16.2% at 3 months, 31.1 ± 16.1% at 6 months, and 25.3 ± 19.7% at 12 months. In the TDR group, mean ODI decreased from 41.4 ± 15.3% at baseline to 28.3 ± 16.3% at 3 months, 23.2 ± 16.1% at 6 months, and 22.9 ± 18.8% at 12 months. No time point showed a statistically significant baseline-adjusted between-group difference. Individual longitudinal analysis (Figure 2B,D,F) showed that the majority of patients in both groups experienced clinically meaningful improvement, defined as a ≥2-point reduction in VAS score or a ≥10-point reduction in ODI score.

### 3.4. Segmental Range of Motion

At 12 months postoperatively, radiographic assessment confirmed successful fusion in all ALIF patients, with no measurable angular motion at the operated segment. In contrast, the TDR group maintained segmental mobility, with a mean range of motion (ROM) of 4.92° (Table 4, Figure 3).

Flexion–extension analysis demonstrated a mean flexion angle of 21.13° ± 7.35° and a mean extension angle of 26.05° ± 8.25° (*p* < 0.0001), yielding a mean ROM of 4.92°. Level-specific analysis showed greater mobility at L4–L5 (mean ROM: 6.9°) than at L5–S1 (mean ROM: 4.17°). The difference between flexion and extension angles was statistically significant at both levels (L4–L5: *p* = 0.0005; L5–S1: *p* < 0.0001), supporting functional motion maintained in the TDR group.

### 3.5. Patient Satisfaction, Medication Use and Quality of Life

Patient satisfaction rates were high in both groups at 12 months postoperatively (Table 5). In the ALIF group, 35 of 38 patients (92.1%) reported being either satisfied or very satisfied (satisfied: 50.0%; very satisfied: 42.1%), with a mean satisfaction score of 75.6 ± 21.0%. In the TDR group, 42 of 49 patients (85.7%) reported being either satisfied or very satisfied (satisfied: 36.7%; very satisfied: 49.0%), with a mean satisfaction score of 73.6 ± 22.6%. Four patients in the TDR group (8.2%) reported a neutral response, while three (6.1%) reported dissatisfaction. Despite these numerical differences, overall satisfaction rates did not differ significantly between groups.

#### 3.5.1. Medication Use

At 12-month follow-up, ongoing pain medication use was reported by 19 of 38 ALIF patients (50.0%) and 21 of 49 TDR patients (42.9%). Among medication users, most relied on simple analgesics or non-steroidal anti-inflammatory drugs (NSAIDs). In the ALIF group, medication use included analgesics alone in 11 patients (28.9%), NSAIDs alone in 3 patients (7.9%), analgesics plus NSAIDs in 3 patients (7.9%), and opioids in 2 patients (5.3%). In the TDR group, medication use included analgesics alone in 11 patients (22.4%), NSAIDs alone in 5 patients (10.2%), analgesics plus NSAIDs in 4 patients (8.2%), and opioids in 1 patient (2.1%). Overall medication use patterns did not differ significantly between groups (*p* = 0.8563).

#### 3.5.2. Return to Work

Successful return to work was achieved by the majority of eligible patients in both groups. In the ALIF cohort, 20 of 33 patients (60.6%) had returned to work by 12 months, with most returning to the same workload as before surgery (18/20, 90.0%) and a minority returning part-time (2/20, 10.0%). In the TDR group, 31 of 43 patients (72.1%) returned to work, with 29 of 31 patients (93.5%) resuming their previous workload and 2 of 31 patients (6.5%) returning part-time. The difference in overall return-to-work rates between groups was not statistically significant (*p* = 0.2908).

### 3.6. Factors Associated with Clinical Outcomes

Correlation analyses were performed to identify demographic, clinical, and procedural factors associated with clinical improvement (Figure 4, Supplemental Figure S2, and Table S1).

#### 3.6.1. Smoking Impact

A multivariable linear regression model of 12-month ODI change (baseline − 12 months; positive = improvement), adjusted for age, sex, BMI, ASA class, and baseline ODI, tested the smoking × surgical-strategy interaction using two operationalizations of smoking status. The binary interaction (any smoker vs. non-smoker × strategy) was borderline and not statistically significant (adjusted coefficient = −12.92, 95% CI −27.41 to +1.58, *p* = 0.085). The dose–response interaction (ordinal smoker code 0–3 × strategy) was highly significant (adjusted coefficient = −9.47, 95% CI −15.64 to −3.30, *p* = 0.004): each additional unit of smoker burden was associated with a 9.5-point smaller ODI improvement in the TDR group than in the ALIF group. Descriptively, higher preoperative smoking burden was associated with reduced ODI improvement in the TDR group (Spearman r = −0.41, 95% CI −0.62 to −0.14, *p* = 0.004) but not in the ALIF group (Spearman r = +0.17, 95% CI −0.16 to +0.46, *p* = 0.32).

#### 3.6.2. Perioperative Variables

In the ALIF group, greater intraoperative blood loss (r = −0.42, *p* < 0.05) and longer hospital stay (r = −0.38, *p* < 0.05) were significantly associated with less ODI improvement at 12 months. These associations were not observed in the TDR group, where perioperative variables showed weak or minimal correlations with postoperative outcomes.

#### 3.6.3. Medication Use and Satisfaction

In both groups, continued analgesic or NSAID use at 12 months was negatively correlated with ODI improvement (ALIF: r = −0.58, *p* < 0.01; TDR: r = −0.61, *p* < 0.001), suggesting that patients with less functional improvement were more likely to require ongoing medication. Conversely, ODI improvement showed strong positive correlations with patient satisfaction (ALIF: r = 0.72, *p* < 0.0001; TDR: r = 0.68, *p* < 0.0001) and return-to-work status (ALIF: r = 0.64, *p* < 0.001; TDR: r = 0.59, *p* < 0.001) in both groups.

## 4. Discussion

- **Principal Findings**

This comparative retrospective cohort study compared short-term clinical outcomes between ALIF and TDR using the LP-ESP prosthesis in 87 patients with symptomatic lumbar DDD. Both surgical approaches produced significant improvements in pain and functional disability at 12-month follow-up. Baseline-adjusted between-group comparisons (ANCOVA) showed a modest early advantage in favor of TDR for VAS low-back pain at 3 and 6 months (below the minimum clinically important difference of 2 points); no statistically significant baseline-adjusted differences were observed for ODI or VAS leg pain at any time point, and the formal treatment × time interaction test was not significant for any outcome. Outcomes converged between groups by 12 months. Complications were infrequent in both groups; the study was not powered to formally demonstrate equivalence of complication rates between the two procedures. Patient satisfaction exceeded 85% in both cohorts, and return-to-work rates were similar (ALIF: 60.6%; TDR: 72.1%). Notably, preoperative smoking was statistically associated with less ODI improvement in the TDR group, whereas perioperative factors, including blood loss and length of hospital stay, were more closely associated with ODI improvement in the ALIF group.

These findings must be interpreted in light of how patients were allocated to each procedure. Treatment selection was not randomized but was instead guided by anatomical and clinical factors, including segmental instability, facet arthropathy severity, spondylolisthesis, and the need for direct neural decompression (Section 2.3). Because these same factors plausibly influence postoperative recovery independently of the surgical strategy chosen, confounding by indication cannot be excluded, and the between-group comparisons reported above should not be read as evidence of comparative effectiveness or clinical equivalence between ALIF and TDR.

- **Comparison with Existing Literature**

Our findings align with and extend the current evidence base comparing fusion and motion-preserving technologies for lumbar DDD. Multiple systematic reviews and meta-analyses have demonstrated that TDR achieves outcomes at least equivalent to fusion, with some studies suggesting superiority in patient-reported outcomes. A 2019 meta-analysis by Bai et al., from randomized controlled trials, found that TDR resulted in significantly better ODI scores and patient-reported outcomes compared to fusion at 2-year follow-up [13]. Similarly, Mu et al. reported superior VAS and ODI scores for TDR versus ALIF at both early (6–12 months) and intermediate (2 years) time points [25].

Our finding of a modest, time-limited VAS low-back pain advantage for TDR—below the minimum clinically important difference and not accompanied by a significant treatment × time interaction—is directionally consistent with, but more conservative than, prior reports of early TDR advantage in second-generation disc prostheses. Lang et al. performed a comparative meta-analysis comparing TDR, anterior stand-alone fusion, and circumferential fusion, demonstrating that TDR provided superior pain relief and function scores at 1-year follow-up, though differences diminished over time [26]. Our baseline-adjusted results support the pattern of subsequent clinical convergence but, given the non-significant treatment × time interaction, do not independently establish a superior early TDR pain-relief effect.

The LP-ESP prosthesis demonstrated clinical performance consistent with other viscoelastic TDR devices. Lazennec et al. reported 7-year outcomes with the LP-ESP, showing mean VAS back pain reductions from 6.5 to 2.8 and ODI improvements from 42% to 18%, with maintained segmental motion (mean ROM 5.3°) [14]. While our 12-month data show more modest improvements (VAS low-back pain from 7.1 to 4.2 and ODI from 41.4% to 22.9%), the overall trajectory was comparable, and our observed mean ROM of 4.92°. Importantly, our complication rates (ALIF 3/38 (7.9%) and TDR 6/49 (12.2%)) compare favorably with published literature. A 2022 systematic review of heterotopic ossification following lumbar TDR reported rates varying from 5% to 60% depending on grading severity [27,28]. We observed no cases of motion-limiting heterotopic ossification at 12 months.

Furthermore a 12-month postoperative assessment was selected as the primary endpoint because randomized controlled trials comparing lumbar total disc replacement with lumbar fusion have consistently demonstrated that the greatest improvements in pain, disability, as assessed by the Oswestry Disability Index (ODI), and health-related quality of life occur during the first postoperative year, with only minimal changes thereafter. This pattern has been reported in the CHARITÉ IDE trial by Blumenthal et al. [29], the ProDisc-L randomized trial by Zigler et al. [30] and its 5-year follow-up by Zigler and Delamarter [31], and was confirmed in the meta-analysis by Wei et al. [32]. Similarly, the LP-ESP lumbar disc prosthesis has demonstrated sustained improvements in pain, disability, and quality of life for up to 5 years after implantation, supporting the concept that longer-term follow-up primarily assesses the durability of clinical outcomes and implant safety rather than additional functional recovery (Lazennec et al., The Spine Journal, 2019) [33].

- **Clinical Implications**

These findings have several important implications for clinical practice. First, both ALIF and TDR represent effective surgical options for appropriately selected patients with symptomatic single-level lumbar DDD refractory to conservative management. The choice between techniques should be individualized based on patient factors, anatomical considerations, and surgeon expertise.

Second, the modest early VAS low-back pain advantage observed with TDR, while below the threshold considered clinically meaningful, may still be relevant for patients who weight early pain relief highly; however, given the non-significant treatment × time interaction, this should not be interpreted as evidence of superior functional recovery, earlier return to work, or reduced indirect costs. For younger, active patients under 45 years—the demographic represented in our cohort, motion preservation offers a theoretical long-term biomechanical rationale in terms of reduced adjacent segment disease risk, a hypothesis that remains unproven and requires validation through extended follow-up.

Third, smoking is a well-established risk factor for poorer outcomes after fusion surgery [17]; available prospective data in lumbar TDR from single-arm cohorts have suggested that smokers can nevertheless achieve improvements in disability and pain comparable to nonsmokers [18]. The mechanism underlying this smoking-specific impact remains unclear and possibilities include compromised bone-implant integration, increased inflammatory response, or impaired soft tissue healing affecting the viscoelastic prosthesis function [34,35]. Our data show a significant dose–response smoking-burden × surgical-strategy interaction (adjusted *p* = 0.004), and preliminary evidence in the interaction analysis reported in Section 3.6.1 supports the hypothesis that TDR outcomes may be more sensitive to preoperative smoking than ALIF outcomes. If confirmed in prospective studies, structured preoperative smoking-cessation programmes could be evaluated as a modifiable risk factor for TDR candidacy.

Fourth, the strong correlation between return-to-work status and overall outcome improvement (r > 0.59, *p* < 0.001 in both groups) underscores the importance of functional recovery beyond pain reduction alone. Successful return to work reflects not only physical improvement but also psychological well-being, social reintegration, and economic independence. Spine surgeons should incorporate RTW as a core outcome measure and consider early engagement with occupational therapists and vocational rehabilitation specialists to optimize this outcome [36,37].

- **Novelty of the Present Study**

The general comparison of TDR with fusion has already been addressed by prior randomized trials and meta-analyses. The present study is intended to be complementary to, rather than duplicative of, this literature, on three specific points. First, to our knowledge this is the first direct clinical comparison of ALIF with the LP-ESP viscoelastic prosthesis specifically: prior comparative randomized trials and meta-analyses evaluated first-generation articulating TDR devices (e.g., CHARITÉ, ProDisc-L), whose two- or three-component ball-and-socket bearing design differs mechanically from the LP-ESP’s monobloc viscoelastic construction, so their comparative findings do not necessarily generalize to this device. Second, this study focuses on a homogeneous young, working-age population, in whom the long-term biomechanical consequences of fusion versus motion maintained are potentially most clinically relevant. Third, this study identifies a formal, statistically significant dose–response smoking-burden × surgical-strategy interaction that has not, to our knowledge, been systematically investigated in the prior comparative TDR-versus-fusion literature.

- **Strengths and Limitations**

This study has several notable strengths. This comparative retrospective cohort design, with multivariable adjustment for prespecified confounders, sought to reduce the influence of measured between-group differences. All procedures were performed by experienced spine surgeons using standardized techniques at a single institution, reducing technical variability. Comprehensive outcome assessment included validated patient-reported measures (VAS, ODI), objective radiographic evaluation, functional outcomes (return to work), and patient satisfaction. The homogeneous patient population (age <45 years, single-level pathology, employed) enhances internal validity and provides focused insights for this specific demographic. Finally, the identification of smoking as a TDR-specific predictor represents a novel, hypothesis-generating finding that requires prospective confirmation before it can inform clinical practice.

However, several limitations must be acknowledged. First, the retrospective design and single center setting limit generalizability. The choice between ALIF and TDR was based on clinical and imaging findings rather than randomization (Section 2.3), and confounding by indication related to unmeasured factors cannot be excluded. In particular, the anatomical variables used to guide the choice between ALIF and TDR, including facet arthropathy severity and presence of spondylolisthesis were not included as covariates in the primary ANCOVA adjustment model. Preoperative flexion–extension radiographs were not systematically obtained in the TDR group, so segmental motion at 12 months is described as maintained rather than as evidence of motion preservation from a preoperative baseline. The preservation of segmental motion (mean ROM 4.92°) in TDR patients, while ALIF achieved solid fusion, represents a fundamental biomechanical difference whose long-term clinical significance remains under investigation. The theoretical advantage of motion preservation—namely, reduction in adjacent segment disease by maintaining physiological load distribution—requires validation through longer follow-up studies. Disc degeneration severity was graded using the Pfirrmann classification at baseline and was similar between groups (mean grade 4.4 ± 0.6 in ALIF vs. 4.3 ± 0.6 in TDR); Pfirrmann grade did not correlate with 12-month ODI improvement in either group (ALIF: r = −0.16, *p* = 0.3716; TDR: r = −0.11, *p* = 0.4928; Supplementary Figure S1). Second, the sample size of 87 patients may have limited statistical power for subgroup analyses and rare complications, and the study was not powered to demonstrate equivalence of complication rates between groups. Third, the 12-month follow-up duration, while sufficient to demonstrate early clinical efficacy, is insufficient to assess long-term outcomes such as adjacent segment disease, implant durability, and revision rates. The key advantage of TDR prevention of adjacent segment degeneration typically manifests over 5–10 years, necessitating extended follow-up to validate motion preservation benefits [12,14].

Fourth, comprehensive biomechanical analysis beyond flexion-extension ROM measurement was not performed. Future studies should incorporate advanced imaging techniques to better characterize motion patterns and load distribution. Fifth, cost-effectiveness analysis was not conducted. While we observed similar hospital stays, comprehensive economic evaluation including implant costs, indirect costs, and long-term healthcare utilization is needed. Finally, our cohort consisted exclusively of younger patients with single-level disease and active employment, limiting applicability to older patients or multi-level pathology. Future multicenter studies with broader inclusion criteria are needed to define optimal patient selection criteria across diverse populations.

- **Future Directions**

Several avenues for future research follow from this study. Prospective multicentre studies with longer follow-up (minimum 5–10 years) are needed to confirm the smoking-TDR interaction, to test whether structured preoperative tobacco-cessation programmes can normalize TDR outcomes, and to evaluate long-term motion preservation, adjacent-segment outcomes, implant survival, and cost-effectiveness. Registry-based studies leveraging large administrative databases can provide real-world evidence on rare complications and revision rates. Mechanistic sub-studies incorporating imaging biomarkers of end-plate and adjacent-level disc health, together with biomechanical modelling, may help clarify whether the smoking–TDR association reflects a biological effect or residual confounding.

## 5. Conclusions

In young adults with symptomatic single-level lumbar DDD refractory to conservative management, both ALIF and TDR with the LP-ESP prosthesis produced substantial 12-month improvements in pain, disability, and satisfaction. A modest early difference favoring TDR was observed for VAS low-back pain (below the minimum clinically important difference); no statistically significant between-group differences were demonstrated for any outcome after correction for multiple comparisons. Preoperative smoking burden was associated with reduced ODI improvement specifically in the TDR group, with a statistically significant dose–response smoking-burden × surgical-strategy interaction (*p* = 0.004). The binary version of this interaction (any smoker vs. non-smoker) was not statistically significant (*p* = 0.085) and this finding is sensitive to how smoking exposure is coded. A peri-implant osteointegration mechanism is biologically plausible, drawing on the dental-implant and bone-titanium interface literature, but remains exploratory and hypothesis-generating and is not established by our data. These observational findings warrant evaluation of modifiable preoperative factors, including tobacco use, as candidate criteria for TDR candidacy in prospective studies, but do not yet establish a basis for personalized procedure selection. Confirmation in larger prospective cohorts with longer follow-up is required before these findings inform hard selection criteria.

## Figures and Tables

**Figure 1:**
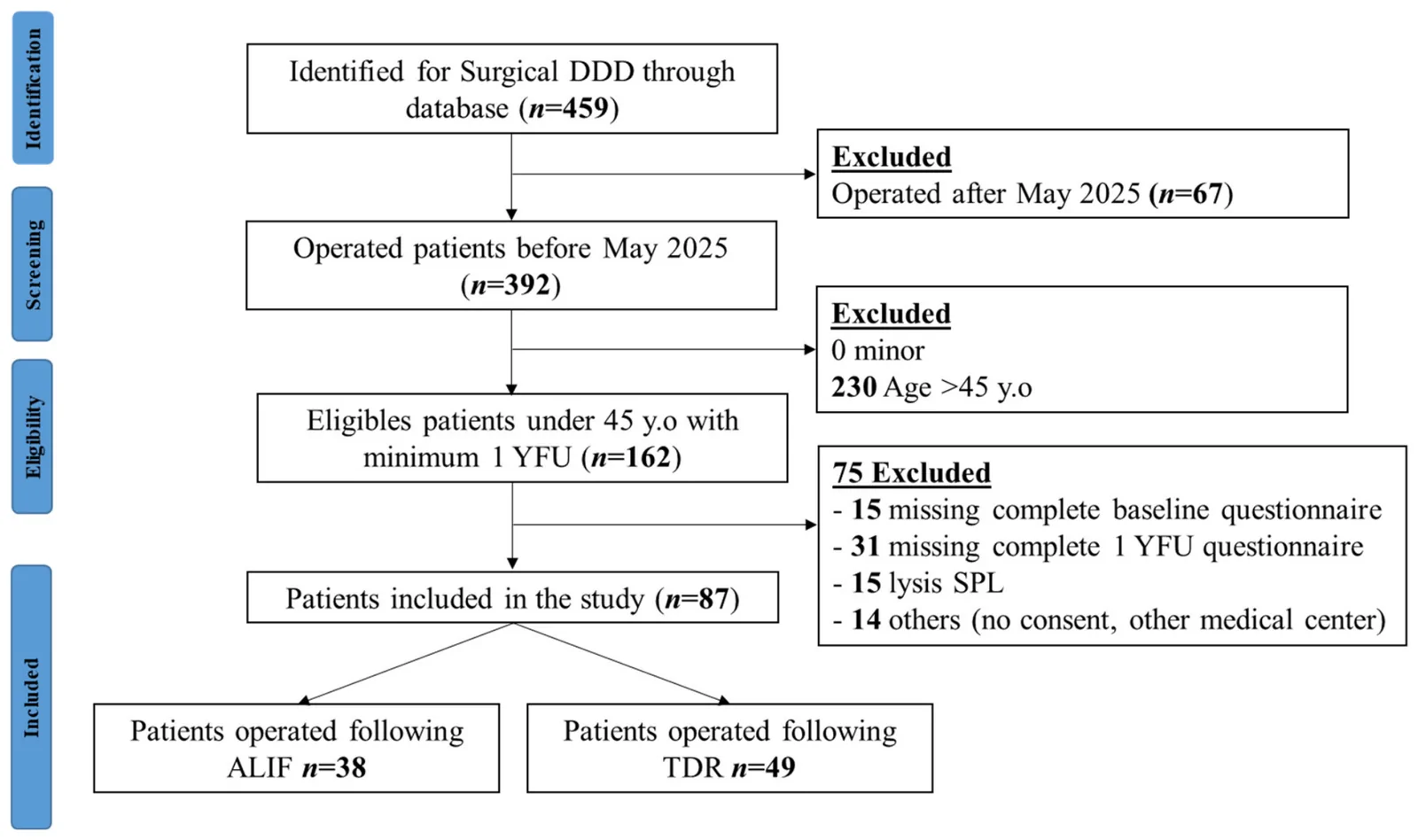
Study FlowChart. Abbreviations: ALIF; Anterior Lumbar Interbody Fusion; TDR; Total disc Replacement; DDD: Degenerative Disc Disease; YFU: Year Follow Up; SPL: Spondylolisthesis.

**Figure 2:**
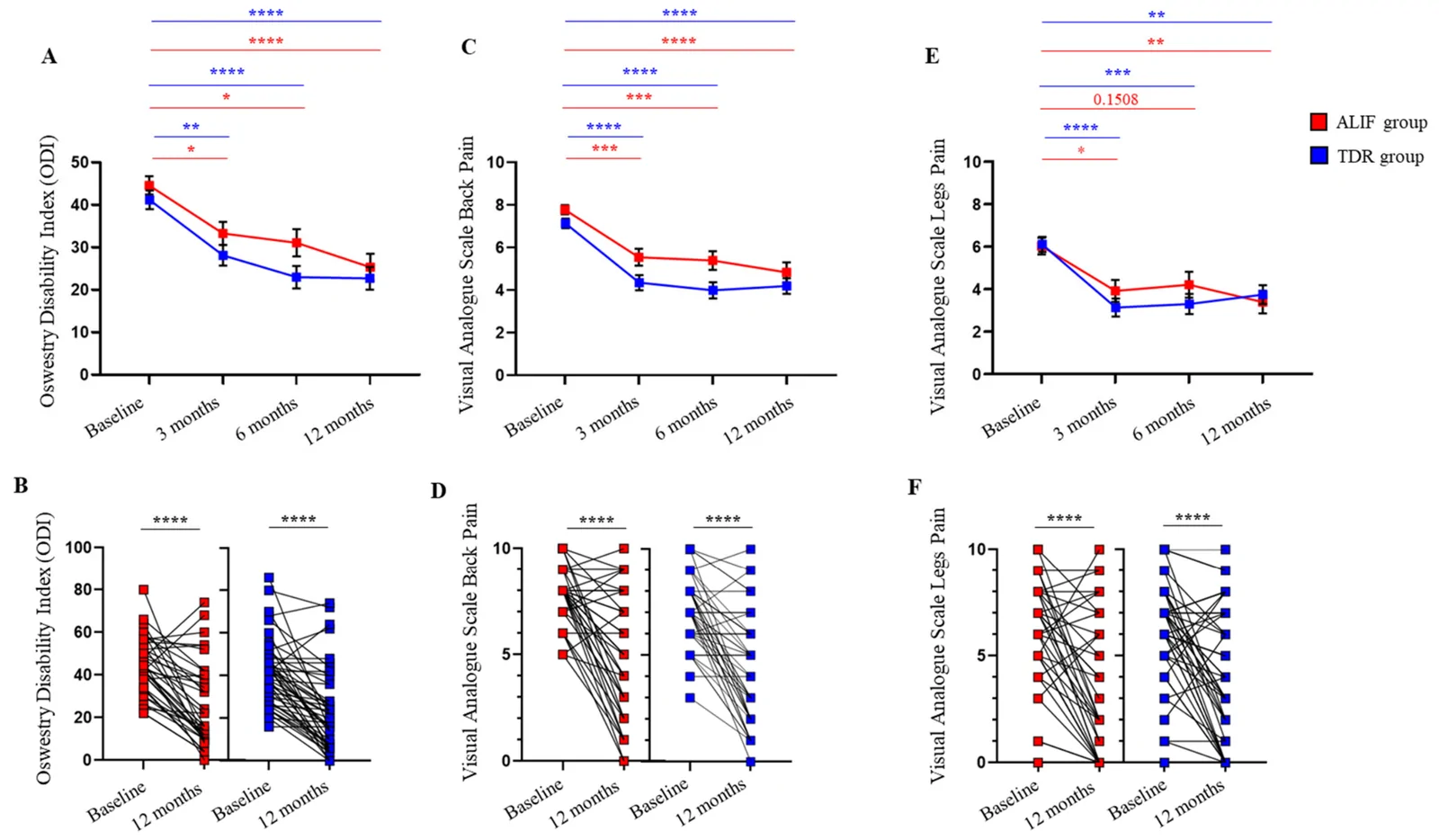
Longitudinal changes in ODI, VAS low-back pain, and VAS leg pain after surgery. Panels (**A**,**C**,**E**) show the percentage change from baseline over time for ODI, VAS low-back pain, and VAS leg pain, respectively. Patient numbers at baseline, 3 months, 6 months, and 12 months were 38, 36, 25, and 38 in the ALIF group and 49, 46, 37, and 49 in the TDR group, respectively. The ALIF group is shown in red and the TDR group in blue. Panels (**B**,**D**,**F**) show the individual patient trajectories from baseline to 12 months for ODI, VAS low-back pain, and VAS leg pain, respectively, including all patients with available baseline and 12-month follow-up data in both groups. (*) correspond to *p* < 0.05, (**) to *p* < 0.01, (***) to *p* < 0.001 and (****) to *p* < 0.0001.

**Figure 3:**
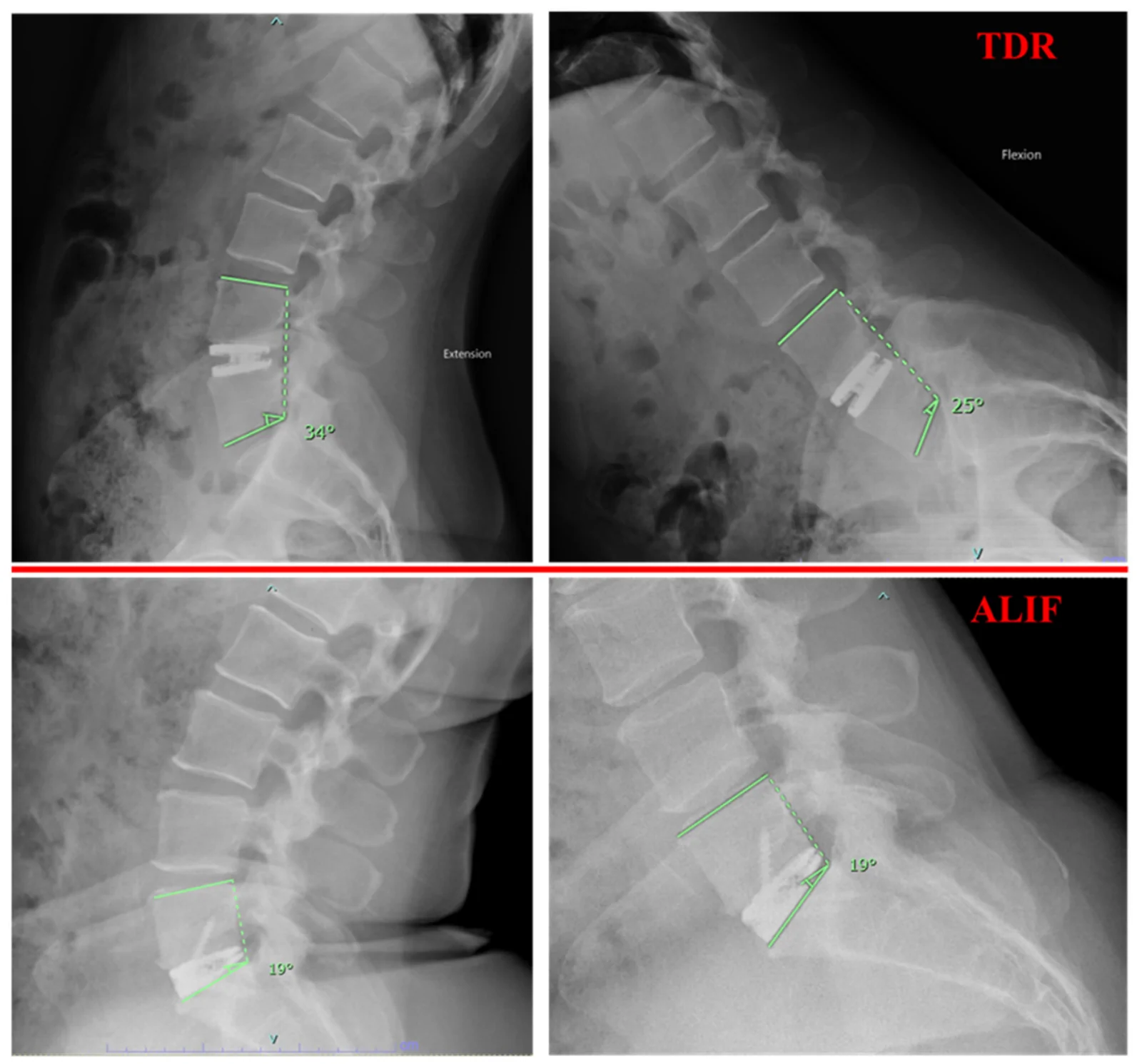
Dynamic flexion–extension radiographs at 12-month follow-up. Segmental angle measurements are shown in flexion and extension for representative TDR patients (**upper panels**) and ALIF patients (**lower panels**).

**Figure 4:**
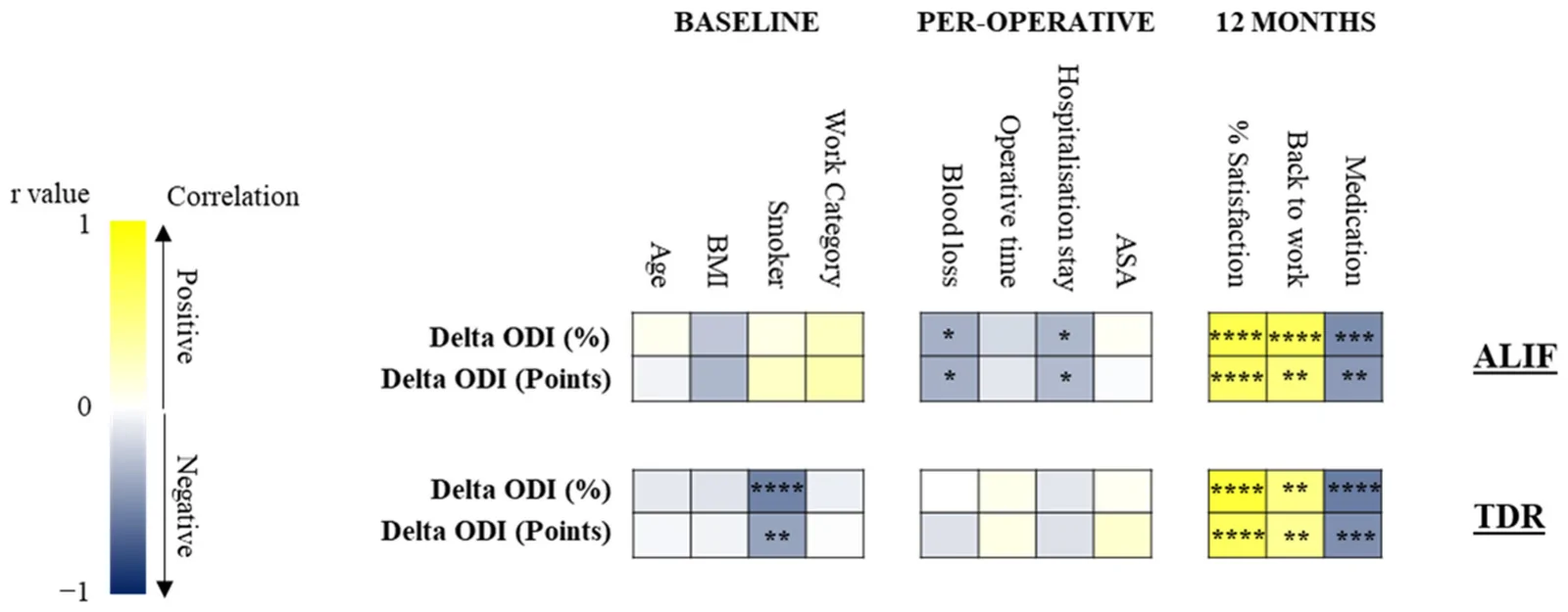
Correlation matrix between change in ODI from baseline to 12-month follow-up and demographic, perioperative, and 12-month follow-up variables for all individual patients. The color gradient, displayed on a blue-to-yellow scale, represents the Spearman’s rank correlation coefficient. Statistically significant correlations are indicated by asterisks: *p* < 0.05 (*), *p* < 0.01 (**), *p* < 0.001 (***), and *p* < 0.0001 (****).

**Table 1:** Patients’ demographic and clinical characteristics. SD: Standard Deviation; ODI: Oswestry Disability Index; VAS: Visual Analogue Scale; ASA: American Society of Anesthestiologists.

Characteristics	ALIF (*n* = 38)	TDR (*n* = 49)	*p*-Value
Sex			
Female	26 (68.4%)	23 (46.9%)	0.0527
Male	12 (31.6%)	26 (53.1%)	
Age (mean, ±SD)	38 ± 5.1	37 ± 5.1	0.3684
Body mass index, kg/m^2^, (mean, ±SD)	27.6 ± 4.8	25.8 ± 4	0.0786
Smoker (*n*, %)	18 (47.4%)	25 (51%)	0.8298
Oswestry Disability Index (ODI), (mean, ±SD)	44.6 ± 13.3	41.4 ± 15.3	0.2050
Low-back pain VAS (scale 0–10), (mean, ±SD)	7.8 ± 1.3	7.1 ± 1.6	0.0341 (*)
Leg-pain VAS (scale 0–10), (mean, ±SD)	6 ± 2.4	6.1 ± 2.5	0.8785
DDD Diagnosis			
Disc herniation	1 (2.7%)	1 (2.1%)	0.1733
Modic type 1 endplate changes	30 (78.9%)	30 (61.2%)	
Both	7 (18.4%)	18 (36.7%)	
DDD Level			
L4L5	11 (28.9%)	15 (30.6%)	>0.9999
L5S1	27 (71.1%)	34 (69.4%)	
ASA			
1	9	20	0.0911
2	22	26	
3	7	3	

(*) correspond to *p* < 0.05.

**Table 2:** Operative and perioperative details.

Operative Details	ALIF	TDR	*p*-Value
Operative time (min) ± SD	64.9 ± 20.3	62.1 ± 18	0.8039
Blood loss (cc) ± SD	63.9 ± 71.6	66.8 ± 66.7	0.9430
Hospital stay (days) ± SD	1.5 ± 1	1.6 ± 1.3	0.7696
Intraoperative complications	1 (2.6%)	0	
Incidental durotomy	1	0	
High estimated blood loss (>500 mL)	0	0	
Cardiopulmonary instability	0	0	
Early postoperative complications	1 (2.6%)	3 (6.1%)	0.6271
Earlier surgical revision	0	0	
Compressive hematoma	1	2	
Sepsis	0	0	
Implant/cage malposition	0	1	
Late postoperative complications	1 (2.6%)	3 (6.1%)	0.6271
Implant loosening	0	2	
Secondary decompression	0	1	
Adjacent segment disease	1	0	

**Table 3:** Longitudinal changes in ODI, VAS low-back pain, and VAS leg pain after surgery in the ALIF and TDR groups. The upper section presents results for the ALIF group, and the lower section presents results for the TDR group. Patient numbers at baseline, 3 months, 6 months, and 12 months were 38, 36, 25, and 38 in the ALIF group, respectively, and 49, 46, 37, and 49 in the TDR group, respectively.

	**ALIF Group**									
	Baseline	3 months	6 months	12 months	Baseline to 3 months	Baseline to 6 months	Baseline to 12 months	3 to 6 months	3 to 12 months	6 to 12 months
**Mean (±SD)**										
ODI (%)	44.6 ± 13.3	33.3 ± 16.2	31.1 ± 16.1	25.3 ± 19.7	0.0288 (*)	0.0153 (*)	<0.0001 (****)	>0.9999	0.2523	>0.9999
VAS low-back pain (%)	7.8 ± 1.3	5.5 ± 2.4	5.4 ± 2.2	4.8 ± 2.9	0.0003 (***)	0.0005 (***)	<0.0001 (****)	>0.9999	>0.9999	>0.9999
VAS leg-pain (%)	6 ± 2.4	3.9 ± 3.1	4.2 ± 3	3.4 ± 3.3	0.0437 (*)	0.1508	0.0018 (**)	>0.9999	>0.9999	>0.9999
	**TDR Group**									
ODI (%)	41.4 ± 15.3	28.3 ± 16.3	23.2 ± 16.1	22.9 ± 18.8	0.0027 (**)	<0.0001 (****)	<0.0001 (****)	0.8749	0.4108	>0.9999
VAS low-back pain (%)	7.1 ± 1.6	4.4 ± 2.4	4 ± 2.3	4.2 ± 2.5	<0.0001 (****)	<0.0001 (****)	<0.0001 (****)	>0.9999	>0.9999	>0.9999
VAS leg-pain (%)	6.1 ± 2.5	3.1 ± 2.9	3.3 ± 2.9	3.7 ± 3.1	<0.0001 (****)	0.0003 (***)	0.0012 (**)	>0.9999	>0.9999	>0.9999

(*) correspond to *p* < 0.05, (**) to *p*< 0.01, (***) to *p*< 0.001 and (****) to *p* <0.0001.

**Table 4:** Treated-segment angle measurements at 12-month follow-up in the TDR group.

	Flexion (°)	Extension (°)	*p*-Value	Flexion-Extension ROM, °, Mean ± SD
All TDR patients ± SD	21.13 ± 7.35	26.05 ± 8.25	<0.0001 (****)	4.92° ± 4.58°
L4-L5, mean ± SD	27.18 ± 8.15	34.09 ± 8.43	0.0005 (***)	6.9° ± 4.52°
L5-S1, mean ± SD	18.83 ± 5.63	23 ± 5.87	<0.0001 (****)	4.17° ± 4.45°

(***) to *p* < 0.001 and (****) to *p* <0.0001.

**Table 5:** Patient-perceived improvement, satisfaction, postoperative pain medication use, and return-to-work status at 12-month follow-up.

	ALIF	TDR	*p*-Value
Patient-perceived improvement, %, mean ± SD	75.6 ± 21	73.6 ± 22.6	0.6799
**Satisfaction, *n* (%)**			0.5436
Very Satisfied	16 (42.1%)	24 (49%)	
Satisfied	19 (50%)	18 (36.7%)	
Neutral/no opinion	1 (2.7%)	4 (8.2%)	
Dissatisfied	2 (5.3%)	2 (4.1%)	
Very dissatisfied	0	1 (2%)	
**Pain medication use, *n* (%)**			0.8563
No medication	19/38 (50%)	28/49 (57.1%)	
Analgesics	11/38 (28.9%)	11/49 (22.4%)	
Non-steroidal anti-inflammatory drugs (NSAIDs)	3/38 (7.9%)	5/49 (10.2%)	
Analgesics + NSAIDs	3/38 (7.9%)	4/49 (8.2%)	
Opioids	2/38 (5.3%)	1/49 (2.1%)	
**Return to work among eligible patients, *n* (%)**	20/33 (60.6%)	31/43 (72,1%)	0.2908
Same workload as before surgery	18/20 (90%)	29/31 (93.5%)	
Part-time return	2/20 (10%)	2/31 (6.5%)	

## Data Availability

The original contributions presented in the study are included in the article; further inquiries can be directed to the corresponding author.

## Supplementary Materials

The following supporting information can be downloaded at https://www.mdpi.com/article/10.3390/jcm15197409/s1, Figure S1: Surgical approach determination based on clinical, morphological, and imaging criteria; Figure S2: Correlation between the change in ODI (ΔODI in percentage) from baseline to 12-month follow-up for all individual patients (ALIF group in red and TDR group in blue); Table S1: Coding of non-numerical variables used in the correlation analysis; Table S2: Comparison of clinical outcomes between the ALIF and TDR groups at each postoperative visit.; Table S3: Case-level detail for the four late postoperative complications; Table S4 Baseline covariate balance between the ALIF and TDR groups, full cohort; Table S5: Propensity-score-matching (PSM) sensitivity analysis; Table S6: Multiple-imputation sensitivity analysis for missing 3- and 6-month follow-up data; Table S7: Minimum clinically important difference (MCID) responder proportions by time point and group; Table S8: Patient attendance at each follow-up time point by group; Table S9: Comparison of baseline characteristics between patients with and without 6-month follow-up data, by treatment group.

## Abbreviations

The following abbreviations are used in this manuscript:

ALIF	Anterior Lumbar Interbody Fusion
TDR	Total Disc Replacement
ODI	Oswestry Disability Index
ASA	American Society of Anesthesiologists
HRQOL	Health-Related Quality of Life Scores
VAS	Visual Analog Scales
DDD	Degenerative Disc Disease
SPL	Spondylolisthesis
BMI	Body Mass Index
ROM	Range Of Motion
SD	Standard Deviation
