# Supplementary material for: Anterior Lumbar Interbody Fusion Versus Total Disc Replacement with a Viscoelastic Prosthesis for Single-Level Lumbar Degenerative Disc Disease in Young Adults: A Comparative Retrospective Cohort Study
Source: J Clin Med. 2026 Sep 24;15(19):7409. doi: 10.3390/jcm15197409 (PMC13665777; doi:10.3390/jcm15197409)
Supplement: Supplementary file 1 [file jcm-15-07409-s001.zip › jcm-4461142-supplementary.pdf]

Supplemental material FIGURE LEGEND

Clinical diagnosis	TDR patients (n=49)	ALIF patients (n=38)
PFIRRMANN	4.3 ± 0.6	4.4 ± 0.6
MODIC 1	36 (73.5%)	31 (81.6%)
ODI vs PFIRRMANN correlation	r = -0.11<br>p = 0.4928	r = -0.16<br>p = 0.3716

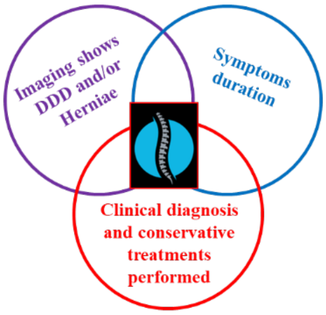

Symptoms duration	TDR Patients (n=49)	ALIF patients (n=38)
Subchronic: 3-12 months	10 (20.4%)	5 (13.2%)
Chronic: > 12 months	39 (79.6%)	33 (86.8%)

Clinical diagnosis	TDR patients (n=49)	ALIF patients (n=38)
**Conservative treatment (Epidural steroid injection)**	39 (79.6%)	30 (78.9%)
**ODI Distribution**		
Minimal disability (0-20)	3 (6.1%)	0
Moderate disability (21-40)	20 (40.8%)	13 (34.2%)
Severe disability (41-60)	21 (42.9%)	22 (57.9%)
Cripple, pain impinges on all aspects of patient's life (61-80)	4 (8.2%)	3 (7.9%)
Patients are bed-bound or exaggerating their symtoms (81-100)	1 (2%)	0

Supplemental Figure S1: Surgical approach determination based on clinical, morphological, and imaging criteria.

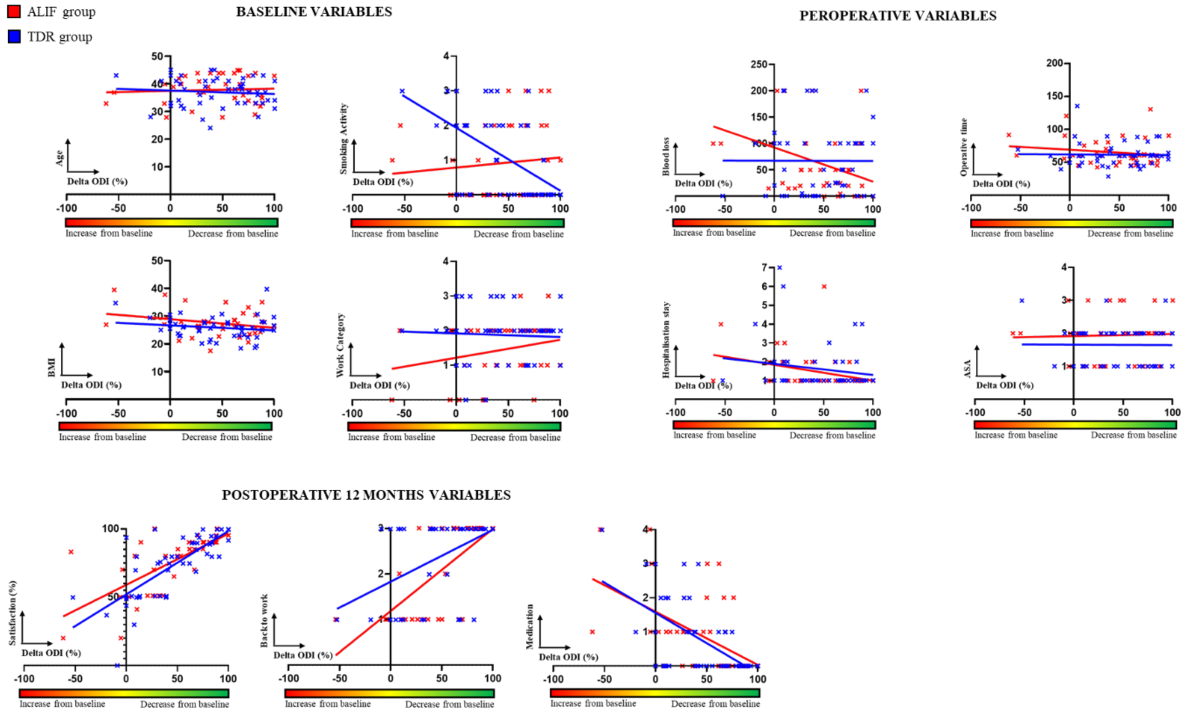

Supplemental Figure S2: Correlation between the change in ODI (ΔODI in percentage) from baseline to 12-month follow-up for all individual patients (ALIF group in red and TDR group in blue). The color gradient (red-to-green scale) in axis representing the decrease ODI (percentage) from baseline (yellow to green) or the increase ODI (percentage) from baseline (yellow to red) at 12 months follow up.

Supplementary Materials — Compiled Tables

Manuscript jcm-4461142 — all supplementary tables referenced

Contents: Table S1 (Coding of non-numerical variables used in the correlation analysis), Table S2 (clinical outcomes comparison), Table S3 (late-complication case detail), Table S4 (baseline covariate balance), Table S5 (PSM sensitivity), Table S6 (multiple-imputation sensitivity), Table S7 (MCID responder proportions), Table S8 (attendance by time point), Table S9 (baseline comparison, present vs missing at 6 months).

Supplementary Table S1

Coding of non-numerical variables used in the correlation analysis.

	Correlation Code	
**Smoking Activity**	No Smoker	0
	Less than 5 & Vapotage	1
	Between 5 to 15	2
	More than 15	3
**Work Category**	No Work	0
	Seated and less than 2 miles per day	1
	Active	2
	Forced laborer	3
**Back to Work**	No	1
	Yes – Half-time	2
	Yes – As before surgery	3
**Medication**	No	0
	Analgesics	1
	Non-Steroidal anti-inflammatory drugs	2
	Both	3
	Opioids	4

Supplementary Table S2

Comparison of clinical outcomes between the ALIF and TDR groups at each postoperative visit.

Outcome (mean %/points ± SD)	ALIF	TDR	P-Value (Mann-Whitney)	Holm-adjusted p
**ODI — 3 months**	33.3 ± 16.2	28.3 ± 16.3	0.2157	1.000
**ODI — 6 months**	31.1 ± 16.1	23.2 ± 16.1	0.0691	0.484
**ODI — 12 months**	25.3 ± 19.7	22.9 ± 18.8	0.6375	1.000
**VAS low-back — 3 months**	5.5 ± 2.4	4.4 ± 2.4	0.0209 *	0.168
**VAS low-back — 6 months**	5.4 ± 2.2	4.0 ± 2.3	0.0187 *	0.168
**VAS low-back — 12 months**	4.8 ± 2.9	4.2 ± 2.5	0.3042	1.000
**VAS leg — 3 months**	3.9 ± 3.1	3.1 ± 2.9	0.1562	0.937
**VAS leg — 6 months**	4.2 ± 3.0	3.3 ± 2.9	0.2681	1.000
**VAS leg — 12 months**	3.4 ± 3.3	3.7 ± 3.1	0.5732	1.000

*Mann–Whitney U test, unadjusted. * p < 0.05. Holm-adjusted p: Holm–Bonferroni correction for the same 9 comparisons (3 outcomes × 3 time points) reported in this table, controlling the family-wise error rate. Computed directly from the 9 raw p-values shown.*

Supplementary Table S3

Case-level detail for the four late postoperative complications (>30 days to 12 months).

Case / Group	Timing after 1rst surgery	Symptoms	Radiographic findings	Management	Revision?	12-mo outcome after first surgery
1/TDR	2 months post op	Recurrence of left-sided sciatica	Slight secondary prosthesis displacement	conservative management assumed — no revision recorded	No	ODI :0<br>VAS leg :0<br>VAS Back pain : 1
2/TDR	8 months post-op	Lower back pain. No radicular pain.	secondary prosthesis displacement	Prosthesis repositioning, then posterior fixation (conversion to instrumented fusion)	Yes — repositioning + fixation	ODI :56<br>VAS leg :6<br>VAS Back pain : 5
3/TDR	10 months post-op	left-sided sciatica-type pain)	slight posterior facet asymmetry with hypertrophy of the left L5-S1 articular mass	Left-sided hemi-decompression (recalibration) at L5–S1	Yes — secondary decompression	ODI :20<br>VAS leg :1<br>VAS Back pain : 3
4/ALIF	8 months post-op	low back pain without radicular radiation	L3-L4 and L2-L3 discopathy with a midline disc protrusion at the L3-L4 level	Anterolateral (LLIF) arthrodesis at L3–L4 and L2–L3 (levels adjacent to the index surgery)	Yes — new fusion, adjacent levels	ODI :54<br>VAS leg :7<br>VAS Back pain : 7

Supplementary Table S4

Baseline covariate balance between the ALIF and TDR groups, full cohort (N = 87), prior to any adjustment or matching.

Covariate	ALIF (n = 38)	TDR (n = 49)	SMD	Balance
**Age, years (mean ± SD)**	37.95 ± 5.11	36.96 ± 5.14	0.193	Modest
**Female sex, n (%)**	26 (68.4%)	23 (46.9%)	0.445	Substantial
**Body mass index, kg/m² (mean ± SD)**	27.63 ± 4.82	25.83 ± 4.05	0.405	Substantial
**Current/former smoker, n (%)**	18 (47.4%)	25 (51.0%)	−0.073	Negligible
**ASA class (ordinal 1–3, mean ± SD)**	1.95 ± 0.66	1.65 ± 0.60	0.470	Substantial
**Segmental level = L4–L5, n (%)**	11 (28.9%)	15 (30.6%)	−0.036	Negligible
**Baseline ODI, % (mean ± SD)**	44.61 ± 13.25	41.37 ± 15.33	0.227	Modest
**Baseline VAS low-back pain**	7.79 ± 1.34	7.14 ± 1.58	0.441	Substantial
**Baseline VAS leg pain**	6.05 ± 2.42	6.10 ± 2.47	−0.020	Negligible

Abbreviations: SD, standard deviation; SMD, standardized mean difference; ASA, American Society of Anesthesiologists physical status class; ODI, Oswestry Disability Index; VAS, visual analog scale.

Balance thresholds: SMD < 0.10, negligible; 0.10–0.25, modest; > 0.25, substantial. Computed directly from the patient-level dataset (n = 38 ALIF, n = 49 TDR).

Supplementary Table S5

Propensity-score matching (PSM) sensitivity analysis.

Methods (computed from the patient-level dataset)

A propensity score for TDR (vs ALIF) was estimated using multivariable logistic regression on age, sex, BMI, ASA class, and smoker code (85 patients with complete data on these five covariates. Patients were matched 1:1, nearest-neighbor on the logit propensity score, without replacement, using a caliper of 0.20 standard deviations of the logit propensity score (caliper = 0.163 logit units, SD of logit PS = 0.812). This yielded 25 matched pairs (50 patients).

Covariate balance before and after matching

Covariate	Pre-match SMD	Post-match SMD (25 pairs)	Balance after
**Age**	0.193	0.043	Negligible
**Female sex**	0.445	−0.16	Modest
**Body mass index**	0.405	0.05	Negligible
**Smoker (any)**	−0.073	−0.161	Modest
**ASA class**	0.470	0.18	Modest

Covariate	Pre-match SMD	Post-match SMD (25 pairs)	Balance after
**Segmental level = L4–L5**	−0.036	0.167	Modest
**Baseline ODI**	0.227	0.078	Negligible
**Baseline VAS low-back pain**	0.441	0.237	Modest
**Baseline VAS leg pain**	−0.020	−0.214	Modest

SMD, standardized mean difference. Balance thresholds: SMD < 0.10, negligible; 0.10–0.25, modest; > 0.25, substantial.

Between-group outcome comparison in the matched cohort (25 pairs, 50 patients)

Outcome (12-month improvement)	ALIF (n=25)	TDR (n=25)	P-value
**ODI, mean ± SD**	20.68 ± 18.73	19.08 ± 18.69	0.884
**VAS low-back pain, mean ± SD**	2.60 ± 3.01	3.32 ± 2.70	0.364
**VAS leg pain, mean ± SD**	2.40 ± 3.25	2.96 ± 3.38	0.598

Mann–Whitney U test, unadjusted (matching itself is the adjustment). Computed directly from the patient-level dataset.

Supplementary Table S6

Multiple-imputation sensitivity analysis for missing 3- and 6-month follow-up data.

Outcome	Time	Complete-case adj Δ (95% CI)	CC p	MI-pooled adj Δ (95% CI)	MI p	n imputed
**Oswestry Disability Index**						
	3 m	−5.08 (−12.76 to +2.61)	0.199	−5.52 (−12.97 to +1.93)	0.146	5
	6 m	−6.34 (−15.05 to +2.37)	0.160	−5.68 (−13.45 to +2.08)	0.151	25
	12 m	+0.52 (−7.19 to +8.22)	0.896	+0.52 (−7.19 to +8.22)	0.896	0
**VAS low-back pain**						
	3 m	−1.46 (−2.63 to −0.30)	0.016	−1.44 (−2.55 to −0.33)	0.011	4
	6 m	−1.35 (−2.63 to −0.07)	0.043	−1.34 (−2.47 to −0.21)	0.020	25
	12 m	−0.30 (−1.53 to +0.92)	0.629	−0.30 (−1.53 to +0.92)	0.629	0
**VAS leg pain**						
	3 m	−1.39 (−2.76 to −0.02)	0.050	−1.36 (−2.71 to −0.00)	0.050	4
	6 m	−1.25 (−2.76 to +0.25)	0.107	−1.18 (−2.63 to +0.26)	0.108	25
	12 m	+0.36 (−0.98 to +1.71)	0.601	+0.36 (−0.98 to +1.71)	0.601	0

Multiple imputation performed under the missing-at-random (MAR) assumption using a regression-based approach with residual perturbation from complete-case residuals, replicated m = 10 times, pooled using Rubin's rules (Rubin DB, Multiple

Imputation for Nonresponse in Surveys, 1987). Imputation model predictors: baseline outcome value, treatment group, age, sex, BMI, smoker status, ASA class. CC, complete case; MI, multiple imputation.

Supplementary Table S7

Minimum clinically important difference (MCID) responder proportions by time point and group.

Time point	ALIF, n/N (%)	TDR, n/N (%)	P-value
**ODI — responder threshold: ≥10-point reduction from baseline**			
**3 months**	16/36 (44.4%)	27/46 (58.7%)	0.2659
**6 months**	13/25 (52.0%)	22/37 (59.5%)	0.6089
**12 months**	29/38 (76.3%)	34/49 (69.4%)	0.6293
**VAS low-back pain — responder threshold: ≥2-point reduction from baseline**			
**3 months**	16/36 (44.4%)	30/47 (63.8%)	0.1184
**6 months**	13/25 (52.0%)	27/37 (73.0%)	0.1103
**12 months**	24/38 (63.2%)	36/49 (73.5%)	0.3542
**VAS leg pain — responder threshold: ≥2-point reduction from baseline**			
**3 months**	18/36 (50.0%)	29/47 (61.7%)	0.3721
**6 months**	13/25 (52.0%)	24/37 (64.9%)	0.4292
**12 months**	22/38 (57.9%)	26/49 (53.1%)	0.6706

Abbreviations: ODI, Oswestry Disability Index; VAS, visual analog scale; MCID, minimum clinically important difference.

Responder = patient whose score improved from baseline to the given time point by at least the MCID threshold shown. Between-group comparisons: Fisher's exact test. Computed directly from the patient-level dataset.

Supplementary Table S8

Patient attendance at each follow-up time point by group.

	ALIF	TDR	P-Value
**Baseline**	38/38 (100%)	49/49 (100%)	> 0.9999
**3 months follow-up**	36/38 (94.7%)	46/49 (93.9%)	> 0.9999
**6 months**	25/38 (65.8%)	37/49 (75.5%)	0.3483
**12 months**	38/38 (100%)	49/49 (100%)	> 0.9999

Values are n (%). Percentages use the total number of patients in each treatment group as the denominator. Differences in follow-up rates between groups were assessed using Fisher's exact test.

Supplementary Table S9

Comparison of baseline characteristics between patients with and without 6-month follow-up data, by treatment group.

ALIF group (25 present vs 13 missing at 6 months):

Characteristic	Present (n=25)	Missing (n=13)	p-value
Age, years	38.12 ± 5.01	37.62 ± 5.50	0.805
Female sex, n (%)	18/25 (72.0%)	8/13 (61.5%)	0.714
Body mass index, kg/m²	26.59 ± 4.28	29.99 ± 5.35	0.069
Current/former smoker, n (%)	12/25 (48.0%)	6/13 (46.2%)	1.000
ASA class (ordinal 1–3)	1.88 ± 0.73	2.08 ± 0.49	0.367
Baseline ODI, %	45.24 ± 10.94	43.40 ± 17.30	0.488
Baseline VAS low-back pain	7.96 ± 1.21	7.46 ± 1.56	0.405
Baseline VAS leg pain	5.92 ± 2.43	6.31 ± 2.46	0.664

TDR group (37 present vs 12 missing at 6 months):

Characteristic	Present (n=37)	Missing (n=12)	p-value
Age, years	37.19 ± 5.16	36.25 ± 5.24	0.649
Female sex, n (%)	16/37 (43.2%)	7/12 (58.3%)	0.508
Body mass index, kg/m²	26.69 ± 3.95	23.19 ± 3.22	0.012
Current/former smoker, n (%)	17/37 (45.9%)	8/12 (66.7%)	0.321
ASA class (ordinal 1–3)	1.68 ± 0.63	1.58 ± 0.51	0.743
Baseline ODI, %	39.23 ± 13.56	47.94 ± 18.99	0.149
Baseline VAS low-back pain	7.14 ± 1.49	7.17 ± 1.90	0.803
Baseline VAS leg pain	6.14 ± 2.32	6.00 ± 2.98	0.887

Continuous variables: Mann–Whitney U test. Categorical variables: Fisher's exact test. Computed directly from the patient-level dataset.
